# Polyunsaturated Fatty Acids Level and Bone Mineral Density: A Two-Sample Mendelian Randomization Study

**DOI:** 10.3389/fendo.2022.858851

**Published:** 2022-07-08

**Authors:** Lin Wang, Chao Zhang, Hao Liang, Nian Zhou, Tianji Huang, Zenghui Zhao, Xiaoji Luo

**Affiliations:** ^1^ Department of Orthopedic Surgery, The First Affiliated Hospital of Chongqing Medical University, Chongqing, China; ^2^ Orthopedic Laboratory of Chongqing Medical University, Chongqing, China

**Keywords:** polyunsaturated fatty acids - PUFA, bone mineral density—BMD, mendelian randomization, osteoporosis, omega - 3 fatty acids

## Abstract

**Background:**

This Mendelian randomization (MR) study aimed to explore the causal relationship between polyunsaturated fatty acids (PUFAs) and bone mineral density (BMD).

**Methods:**

We conducted a two-sample MR analysis to figure out if there is any causal effect of PUFAs on BMD through the summary data from the genome-wide association study (GWAS). Relationships were evaluated through inverse variance weighted (IVW), MR-Egger, weighted median, and maximum likelihood methods. The MR Pleiotropy RESidual Sum and Outlier (MR-PRESSO) test was performed to detect the horizontal pleiotropy.

**Results:**

Our findings revealed that omega-6 fatty acids were negatively related to the TB-BMD (beta-estimate: −0.0515; 95% confidence interval [CI]: −0.0911 to −0.0119; standard error [SE]: 0.0201; p-value: 0.0106). The reverse direction MR analysis showed that TB-BMD was linked to the omega-6 FAs (beta-estimate: −0.0699; 95% CI: −0.1304 to −0.0095; SE: 0.0308; p-value: 0.0265). No statistically significant correlations between PUFAs and BMD were observed after adjusting the interactions between metabolites.

**Conclusion:**

This two-sample MR analyses produced strong and new genomic evidence that there was a causal relationship between omega-6 FAs and BMD. Further investigations are still required to elucidate the potential mechanism.

## Introduction

Osteoporosis is defined as a systematic musculoskeletal disease featured as the loss of bone mass and the degradation of the micro-architecture of the bone tissue, which is invariably predisposed to the increased fragility of bones and incidence of fractures (1, 2). As the global population is aging, it has been considered one of the most pressing public health concerns. According to the statistics, over 9 million osteoporosis-related fractures were confirmed worldwide annually, in which the direct financial losses incurred were estimated at a 17 billion dollars (3). Therefore, osteoporosis now imposes a major economic and clinical burden on society, in addition to inflicting pain and suffering to patients, especially the elderly (4). Nowadays, clinical diagnosis and assessment of osteoporosis rely heavily on bone mineral density (BMD) measurements, which have been proven to be reliable and effective (5, 6). Notably, both osteoporosis and BMD were demonstrated to be highly heritable and polygenic (7–9).

Optimal intake of certain nutrients is proven to participate in the regulation of BMD and is associated with the progress of osteoporosis (10), such as calcium (11) and retinol (12). Of these nutrients, dietary fats were thought to be critical to maintain normal musculoskeletal structure and functions (13–15). As an important component in our dietary fats, fatty acids (FAs) are mainly categorized as long-chain fatty acids including polyunsaturated fatty acids (PUFAs), monounsaturated fatty acids (MUFAs), saturated fatty acids (SFAs), medium-/short-chain fatty acids (MCFAs/SCFAs), and their metabolites (14). According to previous research, PUFAs may have a dual effect on bone metabolism depending on their structure, origin, relative concentration, and metabolic environment (13). In light of this, numerous studies had indicated that omega-6 promotes bone loss, whereas omega-3 favors bone remodeling. Several possible mechanisms had been proposed and clarified (16–18), which include calcium metabolism modulation (19), synthesis of prostaglandin (17), oxidation of fatty acids, genesis of osteoblast (20), and osteoclastogenesis.

Recently, numerous observational studies had indicated a link between PUFAs and BMD, although the findings remain controversial and conflicting. Besides that, observational studies have inherent limitations to infer causal association, such as reverse causality and confounding risk factors.

Mendelian randomization (MR) analyses, which use single nucleotide polymorphisms (SNPs) associated with exposure as instrumental variables (IVs) to evaluate the potentially causal effect between risk factors and outcomes, have evident advantages over conventional observation studies, according to this rationale (21). It is not affected by traditional confounders (environmental exposure and behaviors) and meets the plausibility of causal effect by time order (causes precede effects). A two-sample MR analysis means that IVs associated with exposure and those associated with outcome were obtained from different datasets of population, which could raise the statistical power.

## Methods

To investigate the causal effect of PUFAs on BMD values, we conducted a two-sample MR study that highly relied upon the summary level GWAS data for analysis (22, 23). In a Mendelian randomization analysis, three core assumptions about instrumental variables must be fulfilled: (1) IVs must be strongly related to the exposure, (2) association with the outcome was solely due to the exposure, and (3) independent of any other confounding variables (21). First, we extracted SNPs strongly associated with each PUFAs as instrument values (p<5E−08). To ease the bias due to linkage disequilibrium (LD), we performed the clumping method (R^2^<0.001, window size=10,000 kb). To estimate the degree of LD, the individuals with European ancestors from the 1000 Genomes Project were used as a reference sample (24).

Second, the summary level data of SNPs related to exposure were retrieved in the outcome data.

Third, harmonization of the chosen SNP effect with the risk factors and outcomes was performed to align the palindromic SNPs (with A/T or G/C pairs). Possible palindromic SNPs were excluded.

Following that, we utilized PhenoScanner, a database of human genotype–phenotype associations, to see if any of the chosen SNPs were correlated with the potential confounders for BMD (25, 26). The threshold was set below: genome-wide significance (p<1E−5) and R^2^<0.8. Moreover, F-statistics was used to assess the strength of IVs, and an F-value >10 indicated strong instruments (27). The strength of each instrument was measured by calculating the F-statistic using the following formula: *F* = *R* ^2^(*N* -2)/(1 – *R* ^2^), where R^2^ was the proportion of variance in the phenotype explained by the genetic variants, and N was the sample size (28).

### Genetic Association With PUFAs

The SNP summary data associated with PUFAs were derived from the Nightingale Health UK Biobank Initiative. The UK Biobank recruited 502,639 European participants aged 37–70 years in 22 assessment centers across the UK. All study participants reached the assessment centers by their own means, and enrollment was not performed at nursing homes (29). The biomarker profiles of 500,000 blood samples from UK Biobank were analyzed in Nightingale Health by utilizing nuclear magnetic resonance (NMR) and proprietary software, which could provide over 200 metabolic biomarkers in a single blood test including fatty acids (30). This first release covers biomarker data from approximately 118,000 EDTA plasma samples from baseline recruitment and 5,000 samples from repeat assessment (with 1,500 participants having both baseline and repeat-visit sample in the first data release). The metabolic biomarker dataset was open to any research institutions or individuals *via* the IEU GWAS database, which was a publicly accessible database of genetic correlation from GWAS summary datasets (23).

We only focused on some particular datasets of PUFAs, and three exposure data were selected, namely, omega-3 FAs, omega-6 FAs, and the ratio of omega-6 FAs to omega-3 FAs.

### Genetic Association With BMD

A big GWAS meta-analysis of BMD enrolled 53,236 participants of European origin from GEnetic Factors for Osteoporosis Consortium (31). The femoral neck (FN), the lumbar spine (LS) (L1–L4), and the forearm (FA) were all measured for BMD by dual-energy X-ray absorptiometry (DXA) machines. Each variant with a minor allele frequency (MAF) >0.5% was checked for its effect on BMD, adjusting for sex, age, age^2^, and weight, and standardized to have a mean of zero and standard deviation of one to avoid the potential systematic differences caused by different measuring machines (31). In addition, the summary level data of total body (TB) BMD was employed from one large GWAS meta-analyses comprised of 30 studies and 66,628 individuals from America, Europe, and Australia, in which the majority of the participants came from population-based cohorts of European ancestry (86%) (32). TB-BMD (g/cm^2^) was measured by DXA according to the standard manufacturer protocols. Moreover, its value was corrected for age, weight, height, and genomic principal components (derived from GWAS data), and any additional study-specific covariates (e.g., recruiting center) (32). A detailed information related to the GWAS data is provided and shown in **Supplementary Table S1**.

### Statistical Analyses

We performed the two-sample MR analysis with the inverse variance weighted (IVW) method (23, 33), MR-Egger method (34, 35), weighted median method (36), and maximum likelihood (33) method to estimate the effect of PUFAs for BMD. In algorithm principle, the IVW method might generate the most precise estimate by integrating the Wald ratios of each SNP’s causal effect through meta-analysis (23, 33). To avoid the bias caused by the horizontal pleiotropic effects, we conducted the MR-Egger method and weighted median method to analyze and test the potential directional bias caused by pleiotropy. When no <50% of the weight in the analysis is accounted for by the effective IVs, the weighted median method could offer a plausible estimate of the causal relationships (36). The MR-Egger method, which generated a weighted linear regression between exposure and outcome coefficients, was conducted to evaluate the pleiotropy better. Under the premise of meeting the basic assumption of Instrument Strength Independent of Direct Effect (InSIDE), the slope of the regression line could represent the asymptotically unbiased causal estimate. Apart from this, the horizontal pleiotropy in the average data of the whole genetic instruments could be quantified and presented by the intercept of the MR-Egger regression line (34, 35). Under the condition that the intercept of the regression line is not equal to 0, the intercept of the MR-Egger method can be applied to detect the horizontal pleiotropy. p<0.05 was considered to be statistically significant. Moreover, we also performed multivariable MR (MVMR) analysis to control potential interactions between metabolites. The bidirectional Mendelian randomization was also conducted to explore the reverse causation. All the MR analyses were conducted in R statistical software (Version 4.1.1) by utilizing the “TwoSampleMR” package (https://github.com/MRCIEU/TwoSampleMR) (23).

### Sensitivity Analyses

For sensitivity analysis, several statistics approaches were applied. Cochran Q statistic was tested to assess and quantity heterogeneity (37). Depending on the degree of heterogeneities (Q>0.05 fixed-effect model; Q<0.05 random-effect model), the fixed- or random-effect model was used for further analysis. For quantitative analysis of heterogeneities, we also used I^2^ to evaluate the magnitude. It is generally accepted that I^2^>50% indicates significant heterogeneity. The directional pleiotropy was assessed through the intercept of the MR-Egger method. As a further step, we also conducted the MR Pleiotropy RESidual Sum and Outlier (MR-PRESSO) test to detect the horizontal pleiotropy and remove the outlier SNPs to reassess the cause estimate (38, 39). The “leave-one-out” sensitivity test was applied to figure out the potentially influential single SNP (**Supplementary Figures S1–12**).

## Results

### Causal Effect of PUFA on BMD

**Figure 1:**
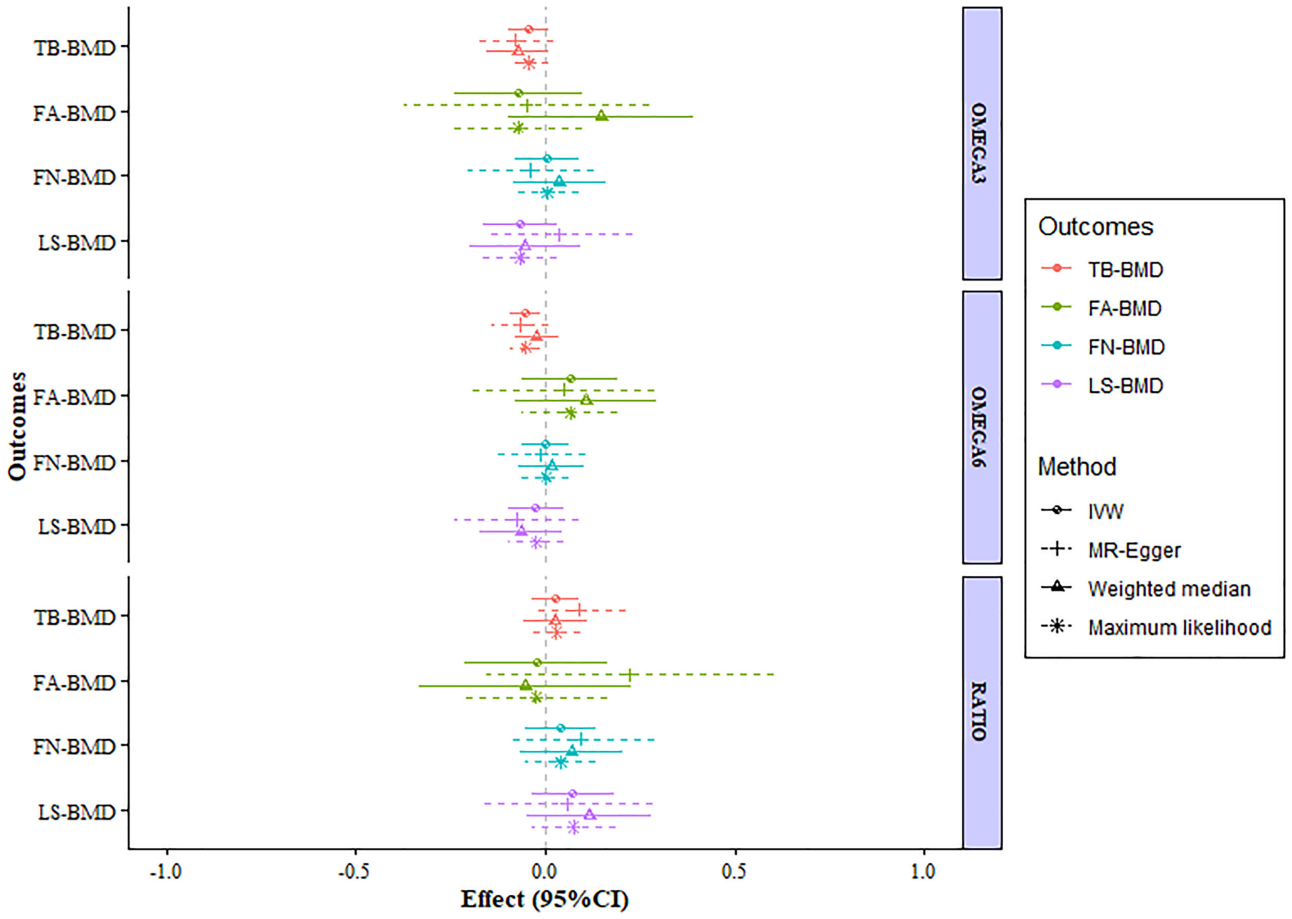
MR estimates of the associations between PUFAs and BMD. The x-axis is the effects of PUFAs on BMD values. The vertical dashed line is the reference at effect = 0. The y-axis presents different BMD types, which are highlighted in different colors. Different MR methods are displayed with different line types. MR, Mendelian randomization; BMD, bone mineral density; FN-BMD, femoral neck BMD; LS-BMD, lumbar spine BMD; FA-BMD, forearm BMD; TB-BMD, total body BMD; SNPs, single nucleotide polymorphisms; IVW, inverse variance weighted; SE, standard error; CI, confidence interval.

After verification, the final data of SNPs enrolled in our analysis are shown in **Supplementary Tables S2–8**. We evaluated the causal effect of PUFA, which includes omega-3 FAs, omega-6 FAs, and the ratio of omega-6 FAs to omega-3 FAs on LS-BMD, FN-BMD, FA-BMD, and TB-BMD in the two-sample MR analysis. The scatter plots are displayed in **Supplementary Figures S13–24**. The results are displayed in **Table 1** and **Figure 1**. Based on the IVW analysis, omega-6 fatty acids were proven to be negatively related to the TB-BMD (beta-estimate: −0.0515; 95% confidence interval [CI]: −0.0911 to −0.0119; standard error [SE]: 0.0201; p-value: 0.0106), which indicated that a 1-SD decrease in omega-6 fatty acids was associated with the improvement in TB-BMD levels by 0.0515 g/cm^2^. The result was further validated by maximum likelihood method (beta-estimate: −0.0517; 95% CI: −0.0915 to −0.0120; SE: 0.0202; p-value: 0.0106). Moreover, no significant correlations were found between omega-6 FAs and site-specific BMD (LS-BMD, FN-BMD, and FA-BMD) according to the statistical analysis results of IVW method, MR-Egger regression, weighted median method, and maximum likelihood analysis.

**Table 1:** MR estimates of the causal effects of PUFAs on BMD using various analysis methods.

Exposures	Outcomes	Number of SNPs	IVW							MR-Egger						
			Estimate	SE	95%CI	MR p-value	Q-value	Heterogeneity p-value	I²		Estimate	SE	95%CI	MR p-value	Intercept	Intercept P-value
**Omega3**	**LS-BMD**	22	−0.0671	0.0499	−0.1650,0.0307	0.1789	20.5023	0.4896	5%		0.0370	0.0984	−0.1558,0.2299	0.7105	−0.0057	0.2334
	**FN-BMD**	22	0.0041	0.0430	−0.0802,0.0885	0.9237	15.1264	0.8165	5%		−0.0379	0.0849	−0.2044,0.1285	0.6600	0.0023	0.5722
	**FA-BMD**	24	−0.0722	0.0860	−0.2408,0.0963	0.4011	18.2438	0.7441	4.76%		−0.0500	0.1655	−0.3746,0.2744	0.7651	−0.0012	0.8769
	**TB-BMD**	36	−0.0438	0.0265	−0.0958,0.0082	0.0989	45.0938	0.1180	2.94%		−0.0817	0.0541	−0.1878,0.0243	0.1403	0.0023	0.4040
															
**Omega6**	**LS-BMD**	29	−0.0247	0.0371	−0.0975,0.0481	0.5058	38.9516	0.0817	3.70%		−0.0753	0.0835	−0.2390,0.0884	0.3752	0.0033	0.4814
	**FN-BMD**	29	0.0008	0.0319	−0.0618,0.0633	0.9812	12.8553	0.9935	3.70%		−0.0116	0.0602	−0.1296,0.1064	0.8482	0.0008	0.8101
	**FA-BMD**	30	0.0646	0.0650	−0.0628,0.1919	0.3205	22.8406	0.7838	3.57%		0.0476	0.1229	−0.1933,0.2885	0.7015	0.0011	0.8722
	**TB-BMD**	45	−0.0515	0.0201	−0.0911, −0.0119	0.0106	41.8432	0.5644	2.33%		−0.0660	0.0376	−0.1398,0.0077	0.0864	0.0010	0.6503
															
**Ratio of Omega6 to Omega3**	**LS-BMD**	18	0.0726	0.0562	−0.0376,0.1829	0.1966	14.2175	0.6516	6.25%		0.0566	0.1156	−0.1701,0.2833	0.6312	0.0009	0.8759
	**FN-BMD**	18	0.0395	0.0482	−0.0550,0.1342	0.4124	13.4092	0.7083	6.25%		0.0933	0.0994	−0.1014,0.2882	0.3616	−0.0030	0.5446
	**FA-BMD**	20	−0.0243	0.0965	−0.2134,0.1648	0.8010	17.1364	0.5806	5.56%		0.2241	0.1925	−0.1532,0.6015	0.2595	−0.0144	0.1532
	**TB-BMD**	26	0.0283	0.0318	−0.0341,0.0909	0.3734	27.3838	0.3369	4.17%		0.0895	0.0633	−0.0345,0.2136	0.1699	−0.0039	−0.2676
Exposures	Outcomes	Number of SNPs	Weighted median							Maximum likelihood					
			Estimate		SE	95%CI		MR p-value			Estimate		SE		95%CI
**Omega3**	**LS-BMD**	22	−0.0539		0.0743	−0.1996,0.0917		0.4680			−0.0675		0.0502		−0.1659,0.0309
	**FN-BMD**	22	0.0367		0.0622	−0.0851,0.1587		0.5544			0.0041		0.0432		−0.0805,0.0888
	**FA-BMD**	24	0.1477		0.1248	−0.0970,0.3924		0.2369			−0.0726		0.0864		−0.2420,0.0966
	**TB-BMD**	36	−0.0727		0.0414	−0.1540,0.0085		0.0795			−0.0444		0.0267		−0.0968,0.0079
										
**Omega6**	**LS-BMD**	29	−0.0639		0.0552	−0.1721,0.0444		0.2475			−0.0246		0.0373		−0.0978,0.0486
	**FN-BMD**	29	0.0156		0.0445	−0.0716,0.1027		0.7261			0.0008		0.0320		−0.0619,0.0635
	**FA-BMD**	30	0.1069		0.0946	−0.0785,0.2922		0.2584			0.0651		0.0652		−0.0626,0.1928
	**TB-BMD**	45	−0.0229		0.0293	−0.0804,0.0345		0.4338			−0.0517		0.0202		−0.0915,−0.0120
										
**Ratio of Omega6 to Omega3**	**LS-BMD**	18	0.1160		0.0836	−0.0479,0.2799		0.1654			0.0736		0.0565		−0.0372,0.1844
	**FN-BMD**	18	0.0692		0.0694	−0.0668,0.2054		0.3184			0.0399		0.0484		−0.0550,0.1350
	**FA-BMD**	20	−0.0520		0.1420	−0.3305,0.2263		0.7138			−0.0245		0.0970		−0.2146,0.1656
	**TB-BMD**	26	0.0260		0.0436	−0.0595,0.1115		0.5510			0.0288		0.0321		−0.0341,0.0917

MR, Mendelian randomization; BMD, bone mineral density; FN-BMD, femoral neck BMD; LS-BMD, lumbar spine BMD; FA-BMD, forearm BMD; TB-BMD, total body BMD; SNPs, single nucleotide polymorphisms; IVW, inverse variance weighted; SE, standard error; CI, confidence interval.

A higher ratio of omega-6 FAs to omega-3 FAs was proven to be poorly related to the improved BMD of the lumbar spine (beta-estimate: 0.0726; 95% CI: −0.0376 to 0.1829; SE: 0.0562; p-value: 0.1966) (**Table 1**) according to the result of the IVW analysis. Furthermore, this conclusion from IVW approach was in accordance with those of the other three statistical models. Moreover, no significant correlations were found between the ratio of FAs and FN-BMD or FA-BMD according to the statistical analysis results of IVW method, MR-Egger regression, weighted median method, and maximum likelihood analysis.

Analogously, omega-3 fatty acids also demonstrated no positive correlation to LS-BMD (beta-estimate: −0.0671; 95% CI: −0.1650 to 0.0307; SE: 0.0499; p-value: 0.1789), FN-BMD (beta-estimate: 0.0041; 95% CI: −0.0802 to 0.0885; SE: 0.0430; p-value: 0.9237), FA-BMD (beta-estimate: −0.0722; 95% CI: −0.2408 to 0.0963; SE: 0.0860; p-value: 0.4011), and TB-BMD (beta-estimate: −0.0438; 95% CI: −0.0958 to 0.0082; SE: 0.0265; p-value: 0.0989) (**Table 1**). These conclusions above were also further validated by the MR-Egger analysis, weighted median analysis, and maximum likelihood analysis.

### Heterogeneity and Sensitivity Analyses

We analyzed heterogeneity through IVW analysis and applied the MR-Egger regression to analyze the pleiotropy. No heterogeneity for the causal effect of PUFAs on BMD was found in our statistical analysis (e.g., as for the causal effect of omega-3 FAs on LS-BMD: Q=20.5023; heterogeneity p-value=0.4896) (**Table 1**). According to the intercept values from the MR-Egger regression, no directional pleiotropy was detected for the causal effect of PUFAs on BMD (e.g., omega-6 FAs to LS-BMD: intercept=0.0033, intercept p-value=0.4814; for FN-BMD: intercept=0.0008, intercept p-value=0.8101; for FA-BMD: intercept=0.0011, intercept p-value=0.8722) (**Table 1**). The MR-PRESSO global test further validated that both outlier and horizontal pleiotropy were not observed in our MR analyses (e.g., omega-6 FAs: p-value=0.083 to LS-BMD; p-value=0.992 to FN-BMD; p-value=0.783 to FA-BMD) (**Table 2**).

**Table 2:** MR-PRESSO estimates of the causal effects of PUFAs on BMD.

Exposures	Outcomes	Number	Effect	MR p-value	MR-PRESSO
		of			
		SNPs			Global test p-value
**Omega3**	**LS-BMD**	22	−0.079	0.118	0.499
	**FN-BMD**	22	−0.0002	0.995	0.828
	**FA-BMD**	24	−0.075	0.309	0.791
	**TB-BMD**	36	−0.042	0.146	0.174
**Omega6**	**LS-BMD**	29	−0.025	0.577	0.083
	**FN-BMD**	29	0.001	0.972	0.992
	**FA-BMD**	30	0.065	0.272	0.783
	**TB-BMD**	45	−0.0515	0.012	0.541
**Ratio of Omega6 to Omega3**	**LS-BMD**	18	0.05	0.414	0.293
	**FN-BMD**	18	0.029	0.507	0.643
	**FA-BMD**	20	−0.032	0.716	0.638
	**TB-BMD**	26	0.032	0.352	0.317

Among the instrumental variables, MR-PRESSO did not identify any outlier for the causal effect between PUFAs and BMD.

### MVMR and Bidirectional MR

As shown in the result of MVMR (**Table 3**), no statistically significant correlations between PUFAs and BMD were observed after adjusting the interactions between metabolites.

**Table 3:** MVMR result after adjusting the interactions between FAs.

Outcome	Exposures	NumberofSNPs	Effect	SE	MVMR P Value
**LS-BMD**	**Omega-3 fatty acids**	44	−4.612	5.0848	0.3643
	**Omega-6 fatty acids**	47	1.6421	1.8519	0.3752
	**Ratio of omega6/omega3**	28	−4.0504	4.5302	0.3712
**FN-BMD**	**Omega-3 fatty acids**	44	2.3857	3.5399	0.5003
	**Omega-6 fatty acids**	47	−0.8907	1.2893	0.4896
	**Ratio of omega6/omega3**	28	2.1271	3.1539	0.5000
**FA-BMD**	**Omega-3 fatty acids**	44	−6.9028	6.9061	0.3175
	**Omega-6 fatty acids**	48	2.5533	2.5146	0.3099
	**Ratio of omega6/omega3**	28	−6.0379	6.1529	0.3264
**TB-BMD**	**Omega-3 fatty acids**	49	1.1004	3.3400	0.7418
	**Omega-6 fatty acids**	57	−0.4604	1.2164	0.7050
	**Ratio of omega6/omega3**	31	0.9961	2.9757	0.7378

MR, Mendelian randomization; BMD, bone mineral density; FN-BMD, femoral neck BMD; LS-BMD, lumbar spine BMD; FA-BMD, forearm BMD; TB-BMD, total body BMD; SNPs, single nucleotide polymorphisms; IVW, inverse variance weighted; SE, standard error; CI, confidence interval.

As shown in **Tables 4**, **5**, TB-BMD was proven to be negatively related to the omega-6 fatty acids based on the MR-Egger method (beta-estimate: −0.0699; 95% CI: −0.1304 to −0.0095; SE: 0.0308; p-value: 0.0265). No other reverse causations were observed between BMD and PUFAs.

**Table 4:** MR estimates of the causal effects of PUFAs on BMD using various analysis methods.

Exposures	Outcomes	Number of SNPs	IVW								MR-Egger						
			Estimate	SE	95%CI	MR p-value		Q-value	Heterogeneity p-value	I²	Estimate			SE	95%CI	MR p-value	Intercept
**LS-BMD**	**Omega-3 fatty acids**	20	0.0045	0.0151	−0.0251,0.0342	0.7623	24.4995		0.1776	5.56%	−0.0802			0.0635	−0.2048,0.0444	0.2232	0.0062
	**Omega-6 fatty acids**	19	0.0084	0.0201	−0.0311,0.0479	0.6764	29.3822		0.0439	5.88%	−0.0892			0.0723	−0.2310,0.0526	0.2344	0.0071
	**Ratio of omega6/omega3**	20	0.0041	0.0152	−0.0256,0.0341	0.7836	14.7783		0.7365	5.56%	0.0667			0.0576	−0.0463,0.1797	0.2624	−0.0046
														
**FN-BMD**	**Omega-3 fatty acids**	17	−0.0080	0.0234	−0.0544,0.0373	0.7148	27.9430		0.0321	6.67%	−0.1761			0.1192	−0.4099,0.0576	0.1603	0.0108
	**Omega-6 fatty acids**	15	0.0343	0.0251	−0.0150,0.0837	0.1726	25.4820		0.0301	7.69%	0.2845			0.1355	0.0188,0.5501	0.0559	−0.0161
	**Ratio of omega6/omega3**	17	0.0224	0.0178	−0.0125,0.0573	0.2082	17.8123		0.3349	6.67%	0.1857			0.0937	0.0020,0.3694	0.0661	−0.0105
														
**TB-BMD**	**Omega-3 fatty acids**	68	−0.0018	0.0128	−0.0269,0.0233	0.8874	87.3676		0.0481	1.52%	−0.0638			0.0356	−0.1337,0.0060	0.0779	0.0034
	**Omega-6 fatty acids**	68	−0.0007	0.0126	−0.0255,0.0240	0.9515	94.0726		0.0162	1.52%	−0.0699			0.0308	−0.1304,−0.0095	0.0265	0.0041
	**Ratio of omega6/omega3**	68	0.0069	0.0112	−0.0151,0.0291	0.5372	71.5785		0.3284	1.52%	0.0634			0.0324	−0.0002,0.1270	0.0551	−0.0031
Exposures	Outcomes	Number of SNPs	Weighted median								Maximum likelihood						
			Estimate	SE		95%CI	MR p-value				Estimate			SE	95%CI		
**LS-BMD**	**Omega-3 fatty acids**	20	0.0271	0.0225		−0.0171,0.0713	0.2303				0.0046			0.0153	−0.0254,0.0347		
	**Omega-6 fatty acids**	19	0.0231	0.0239		−0.0238,0.0701	0.3343				0.0086			0.0161	−0.0228,0.0402		
	**Ratio of omega6/omega3**	20	0.0035	0.0217		−0.0391,0.0461	0.8712				0.0042			0.0153	−0.0258,0.0343		
										
**FN-BMD**	**Omega-3 fatty acids**	17	−0.0341	0.0262		−0.0856,0.0172	0.1928				−0.0088			0.0179	−0.0441,0.0264		
	**Omega-6 fatty acids**	15	0.0261	0.0284		−0.0297,0.0819	0.3594				0.0355			0.0189	−0.0016,0.0727		
	**Ratio of omega6/omega3**	17	0.0338	0.0254		−0.0159,0.0837	0.1831				0.0231			0.0180	−0.0122,0.0584		
										
**TB-BMD**	**Omega-3 fatty acids**	68	0.0106	0.0178		−0.0243,0.0455	0.5524				−0.0018			0.0113	−0.0241,0.0204		
	**Omega-6 fatty acids**	68	−0.0055	0.0172		−0.0393,0.0281	0.7456				−0.0007			0.0108	−0.0219,0.0203		
	**Ratio of omega6/omega3**	68	0.0115	0.0171		−0.0221,0.0452	0.5001				0.0071			0.0114	−0.0152,0.0294		

MR, Mendelian randomization; BMD, bone mineral density; FN-BMD, femoral neck BMD; LS-BMD, lumbar spine BMD; FA-BMD, forearm BMD; TB-BMD, total body BMD; SNPs, single nucleotide polymorphisms; IVW, inverse variance weighted; SE, standard error; CI, confidence interval.

**Table 5:** MR-PRESSO estimates of the causal effects of BMD on PUFAs.

Exposures	Outcomes	Number	Effect	MR p-value	MR-PRESSO
		of			
		SNPs			Global test p-value
**LS-BMD**	**Omega-3 fatty acids**	20	−0.001	0.933	0.207
	**Omega-6 fatty acids**	19	0.003	0.845	0.034(no outlier)
	**Ratio of omega6/omega3**	20	0.008	0.487	0.817
**FN-BMD**	**Omega-3 fatty acids**	17	−0.008	0.719	0.035(no outlier)
	**Omega-6 fatty acids**	15	0.034	0.194	0.049(no outlier)
	**Ratio of omega6/omega3**	17	0.022	0.250	0.389
**TB-BMD**	**Omega-3 fatty acids**	68	−0.006	0.619	0.075
	**Omega-6 fatty acids**	68	−0.001	0.882	0.013(no outlier)
	**Ratio of omega6/omega3**	68	0.011	0.326	0.391

## Discussion

The PUFAs contain two main acid types: omega-3 and omega-6 FAs. Omega-3 PUFAs are a group of fatty acids mainly synthesized in the body and maintained through diet, which predominantly include eicosapentaenoic acid (EPA), alpha-linolenic acid (ALA), and docosahexaenoic acid (DHA). Correspondingly, omega-6 fatty acids that are mainly found in various vegetable oils always come from linoleic acid (LA).Recently, several observational studies reported conflicting and discrepant conclusions on the association between PUFAs and BMD. Therein, omega-3 FAs were validated to positively affect bone remodeling *via* many different processes, including inhibiting osteoclast and promoting osteoblast activities. On the contrary, omega-6 FAs were always thought to be proinflammatory and pernicious to the maintenance of bone health. Accumulating animal experiments have revealed that supplementation of omega-3 FAs could enhance bone density and improve bone quality by various mechanisms. Acting as the specific ligand of peroxisome proliferator-activated receptor γ (PPAR γ), PUFAs could bind to the PPAR γ and induce the differentiation of adipocytes and fatty acids metabolism, which in turn affect the metabolism of the bone tissue (13). In addition, PUFAs could also modulate the formation of inflammatory cytokines to regulate the balance between formation and resorption of the bone *via* acting on the biosynthetic pathway of prostaglandin E2 (PGE2). Previous studies revealed that PUFAs could regulate the expression or the enzyme activity of cyclooxygenase (COX)-2, which is a rate-limiting enzyme in the synthesis of PGE2. The dual effect of PUFAs is that omega-3 FAs favor the downregulation of COX-2, which leads to the decrease in the production of PGE2 and, furthermore, enhance the formation of the bone. As for omega-6 FAs, it produced the entire opposite effect. PUFAs could also affect the bone marrow microcirculation to reduce the metabolic capacity of the bone. The effect of promoting uptaking of calcium from diet has also been reported. Inconsistent with the conclusion drawn from animal models, the observations from clinical trials still remain controversial.

To the best of our knowledge, this is the first time that the causal association between PUFAs and BMD through a two-sample MR analysis is investigated. Our analysis involved 53,236 individuals of European decent for the association with site-specific BMD, 66,628 individuals for TB-BMD, and 114,999 individuals for PUFAs. Our analytical studies demonstrated that omega-6 fatty acids were proven to be negatively related to the TB-BMD. Moreover, reverse causation was also observed between them. However, after adjusting the interactions between metabolites, no cause and effect association was shown based on the MVMR result. This may suggest that the associations between PUFAs and BMD are likely contributed by other confounding risk factors or the interactions between FAs. To ensure the consistency and reliability of the analysis, our research employed multiple statistical process to check the heterogeneity and control the pleiotropy. We also selected the IVs (F-statistics>10) from the large GWAS data to better represent PUFAs and BMD. In general, our two-sample MR study possessed adequate precision and stability to support the conclusion.

As far as we know, the previous observational studies were always limited to the effect of some specific types of PUFAs on bone health or some particular subtypes of the population, such as post-menopausal women and older people. Furthermore, the intake of dietary fatty acids was usually retrospectively estimated using some questionnaires (40, 41). Thus, the inherent methodological limitation of evaluating the supplementation of fatty acids is unavoidable (42). Due to this, it is not surprising that the previous studies are controversial while still puzzling. Most of the observational studies found that BMD was positively correlated with the supplementation of omega-3 PUFA or fish oil. According to the Women’s Health Initiative Study, positive associations between hip fractures and omega-3 FAs were shown; however, inverse associations were observed between omega-6 FAs, MUFAs, and PUFAs (43). Similarly, in a study of 76,000 women and 45,000 men enrolled, the fracture risk was negatively correlated with the consumption of omega-6 FAs and PUFAs (40). In contrast, a few researchers reported no statistically significant relationship between consumption of PUFAs and BMD or the incidence of fracture (44, 45). Other studies observed completely different findings in which a higher intake of PUFAs may deteriorate bone loss (43). In a recent meta-analysis that enrolled 28 RCTs (7,288 participants), the experimenter reported that the increased supplementation of omega-3 FAs may exert a low magnitude to the increase in BMD of the lumbar spine by 2.6% and femoral neck by 4.1%; however, the grade of evidence was insufficient (46). Another interesting finding that emerged from the analysis is that the increasing intake of total PUFAs may have little to no effect on BMD (46). Recently, in a single-center study of postmenopausal Spanish women, a high level of plasma omega-3 FAs was an independent risk factor of bone health (47).

The vast discrepancy of various studies may be attributed to multiple complex confounders such as sex and gender, etc. One notable confounder is that the consumption of cod liver oil rich in vitamins A and D was likely to exert influence to some degree on bone health (48, 49). Some researchers attempt to explain this phenomenon with more objective and more profound mechanisms, such as circulating fatty acids. Based on the Framingham Osteoporosis Study, which included 765 participants, a negative trend was observed between arachidonic acid (AA) and risk of hip fracture (50). Another cross-sectional study indicated that greater red blood cell omega-3 FAs were beneficial to decrease the risk of hip fracture (51). One important finding is that the influence of fatty acids on BMD may vary dynamically over time, beyond possible sex differences (42).

In accumulating animal experiments, the mechanism of PUFAs affecting bone health could be better elaborated. Deep down to the microlevel, the benefits of fish oil is closely linked to the presence of allelic variants in some genes such as PPAR γ, according to a comparative study on mice with polymorphisms in the PPAR γ gene (6T) (52). On the contrary, no effect of consumption of PUFAs on bone structure or metabolism was found in healthy mice. In another study conducted in ovariectomized rats, the level of PUFAs and ratio of omega-6/omega-3 PUFAs could be the essential important factors for maintaining BMD and bone turnover markers (53). The dietary ratio of 5:1 significantly elevated the amount of DHA in the bone tissues of the femur. This conclusion was also supported by some observational population study; in an investigated population with a higher intake ratio of omega-3 FAs to omega-6 FAs, such as the Japanese population, a lower ratio of osteoporosis was reported (54). In addition, different dietary sources of omega-3 FAs exhibited significant disparities in biochemistry and metabolism. Rozner et al. found that flaxseed oil was effective in ameliorating the micro-architecture, and fish oil could improve BMD, in which the core mechanism may be the alteration of peripheral clock in bone cells (55).

Some study limitations should be noted, although the rationale of MR analyses made it superior to conventional observational studies in excluding the existence of confounders. First, we only focused on the causal associations between a specific type of PUFA and BMD and did not take into consideration some other nutrients that might interact with PUFAs and cause bias. The potential limitation might contribute to the implausible casual relationship between PUFAs and BMD to some extent. Therefore, we conducted MR-Egger and MR-PRESSO methods to exclude the potential pleiotropy. Furthermore, the PhenoScanner tool was adopted to screen and remove the SNPs associated with confounders. Hence, the conclusion of this study should be creditable. Second, the samples were not further substratified according to gender and age, which were believed to be important risk factors of BMD based on previous studies. However, the effect on our analyses could be small due to the strength of the large sample size. Lastly, the exact mechanism underlying the causality between them was not explored in-depth. Therefore, a mechanistic research should be carried out in the future.

## Conclusion

This two-sample MR analysis produced strong and new genomic evidence that there was causal relationship between omega-6 FAs and BMD. However, a further validation by MVMR and bidirectional MR suggested that the association between them may be caused by the interactions of metabolites and reverse causality. Further investigations are still required to elucidate the potential mechanism.

## Data Availability Statement

The original contributions presented in the study are included in the article/**Supplementary Material**, further inquiries can be directed to the corresponding author/s.

## Author Contributions

LW and XL conducted study design. LW, CZ, and HL conducted data collection and statistical analysis. LW, NZ, TH, and ZZ conducted data interpretation, manuscript preparation, and literature search. LW and XL conducted funds collection. All authors contributed to the article and approved the submitted version.

## Funding

This study was funded by the National Natural Science Foundation of China (81873998).

## Conflict of Interest

The authors declare that the research was conducted in the absence of any commercial or financial relationships that could be construed as a potential conflict of interest.

## Publisher’s Note

All claims expressed in this article are solely those of the authors and do not necessarily represent those of their affiliated organizations, or those of the publisher, the editors and the reviewers. Any product that may be evaluated in this article, or claim that may be made by its manufacturer, is not guaranteed or endorsed by the publisher.
