# Supplementary material for: Polyunsaturated Fatty Acids Level and Bone Mineral Density: A Two-Sample Mendelian Randomization Study
Source: Front Endocrinol (Lausanne). 2022 Jul 8;13:858851. doi: 10.3389/fendo.2022.858851 (PMC9304696; doi:10.3389/fendo.2022.858851)
Supplement: Supplementary file 1 [file DataSheet_1.docx]

**
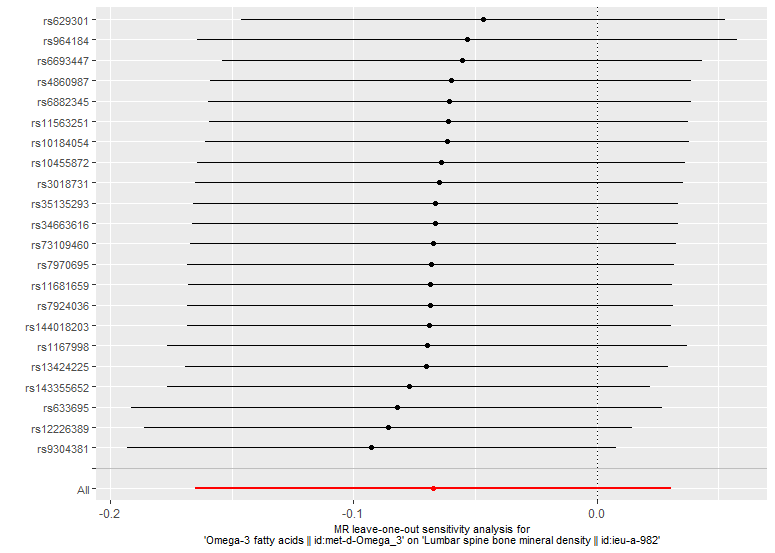
**

**Figure S1.** The “leave-one-out” sensitivity test for omega-3 fatty acids on LS-BMD.


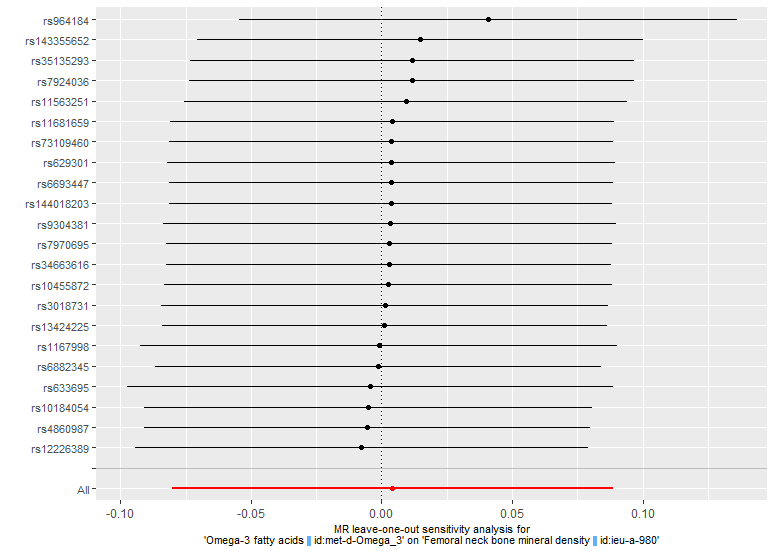


**Figure S2.** The “leave-one-out” sensitivity test for omega-3 fatty acids on FN-BMD.


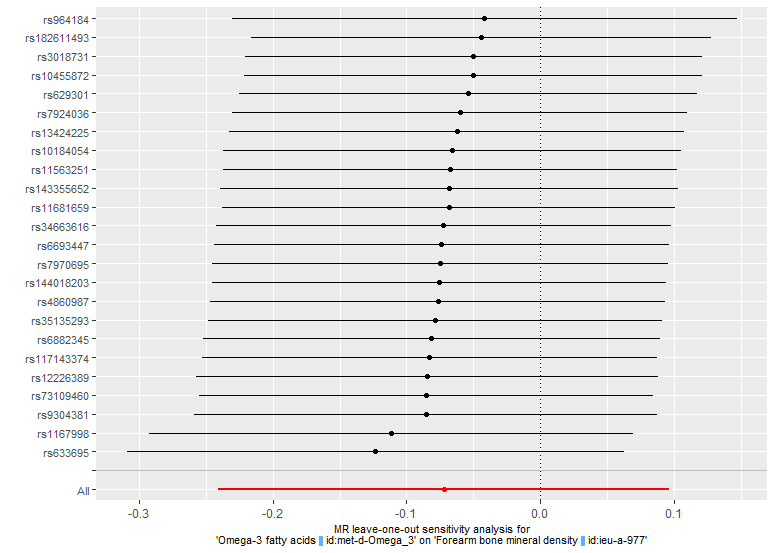


**Figure S3.** The “leave-one-out” sensitivity test for omega-3 fatty acids on FA-BMD.


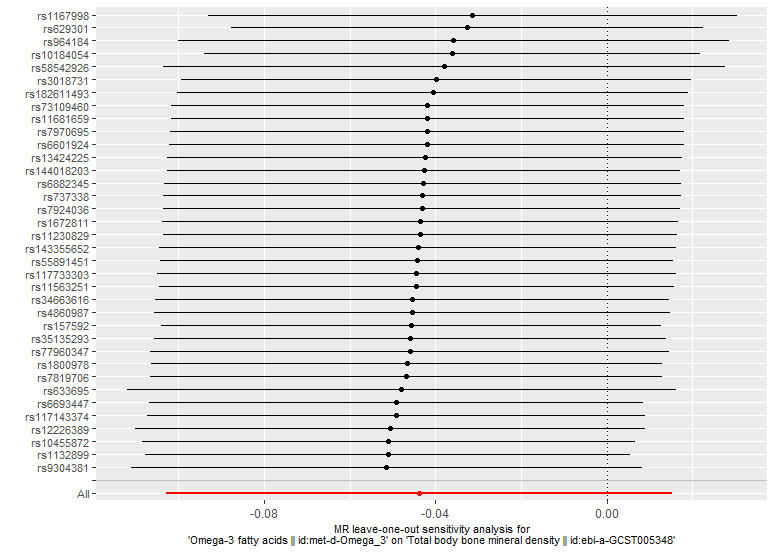


**Figure S4.** The “leave-one-out” sensitivity test for omega-3 fatty acids on TB-BMD.


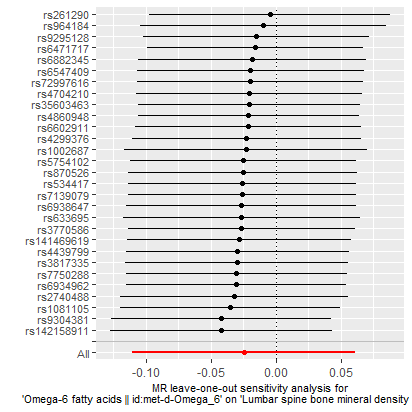


**Figure S5.** The “leave-one-out” sensitivity test for omega-6 fatty acids on LS-BMD.


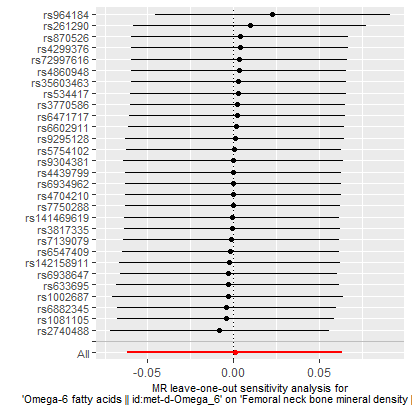


**Figure S6.** The “leave-one-out” sensitivity test for omega-6 fatty acids on FN-BMD.


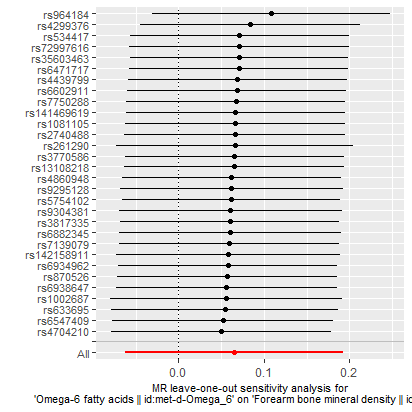


**Figure S7.** The “leave-one-out” sensitivity test for omega-6 fatty acids on FA-BMD.


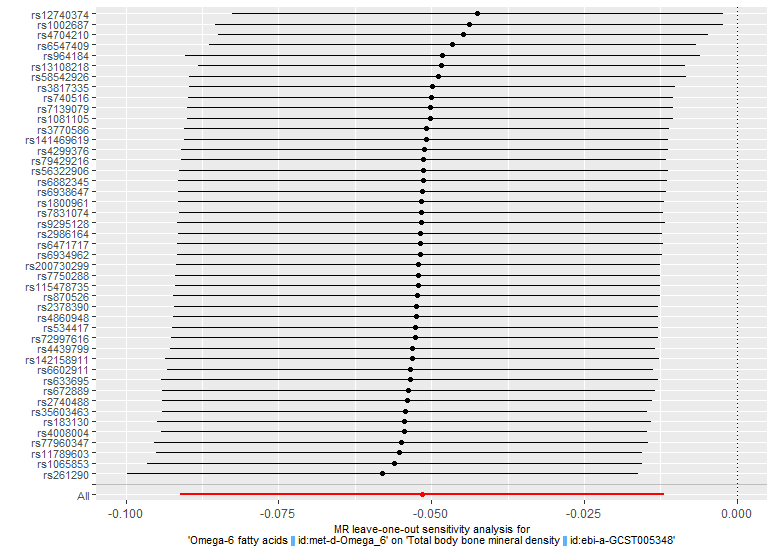


**Figure S8.** The “leave-one-out” sensitivity test for omega-6 fatty acids on TB-BMD.


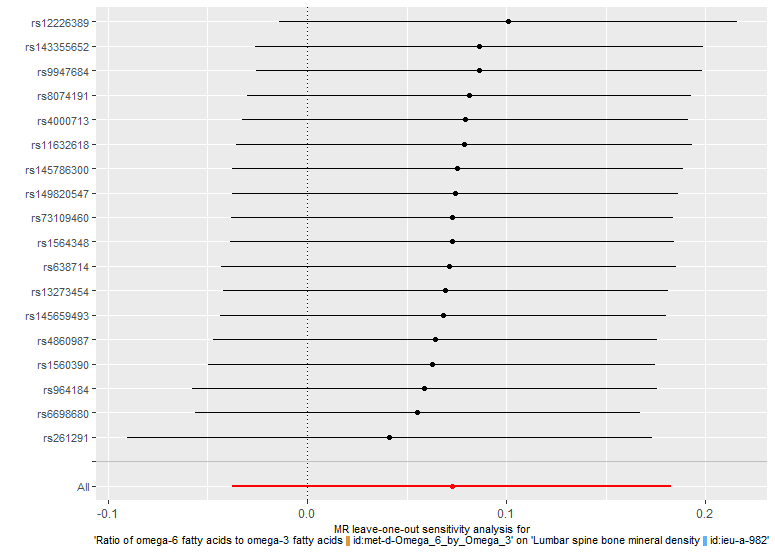


**Figure S9.** The “leave-one-out” sensitivity test for Ratio of omega-6 fatty acids to omega-3 fatty acids on LS-BMD.


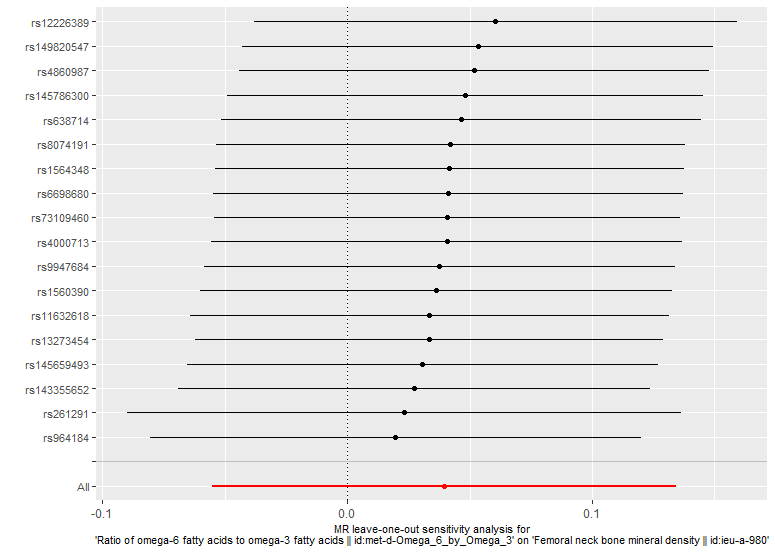


**Figure S10.** The “leave-one-out” sensitivity test for Ratio of omega-6 fatty acids to omega-3 fatty acids on FN-BMD.


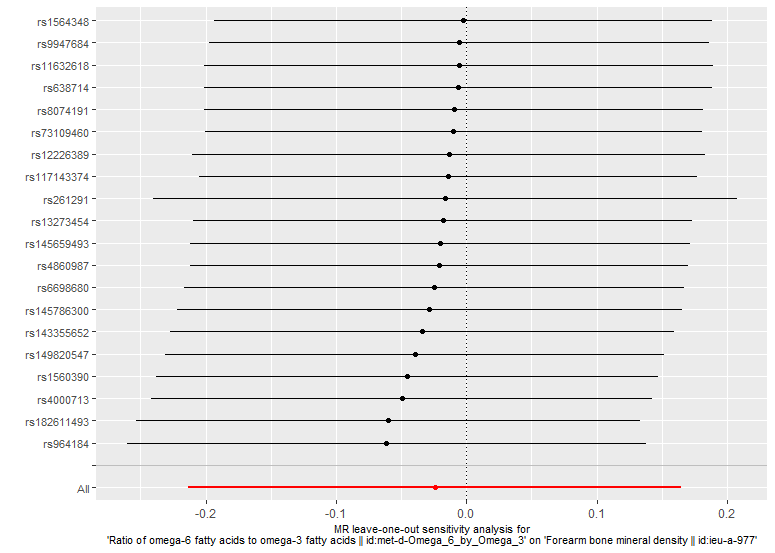


**Figure S11.** The “leave-one-out” sensitivity test for Ratio of omega-6 fatty acids to omega-3 fatty acids on FA-BMD.


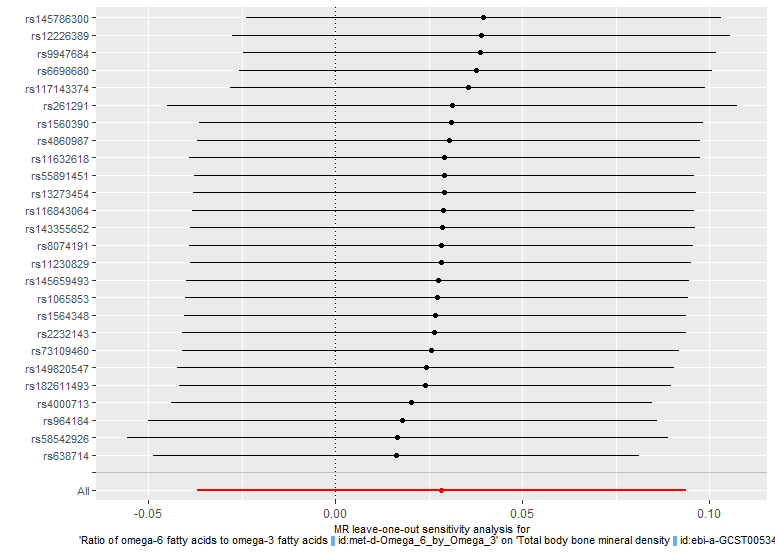


**Figure S12.** The “leave-one-out” sensitivity test for Ratio of omega-6 fatty acids to omega-3 fatty acids on TB-BMD.


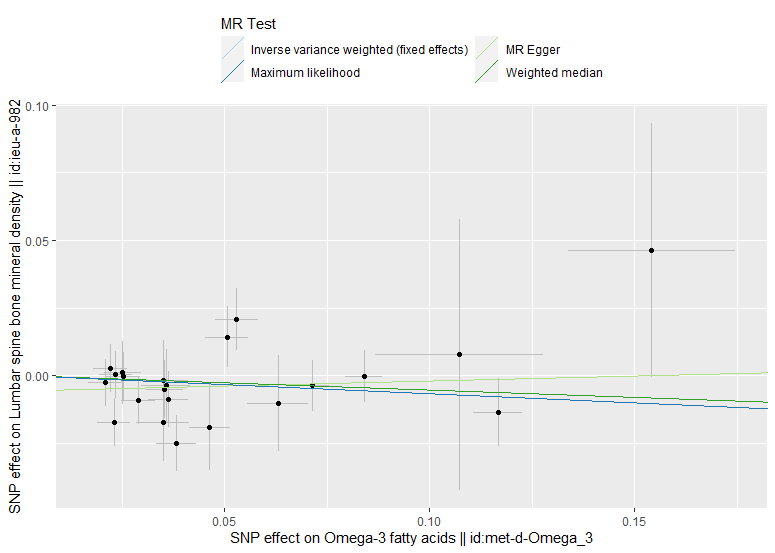


**Figure S13.** The scatter plot for omega-3 fatty acids on LS-BMD.


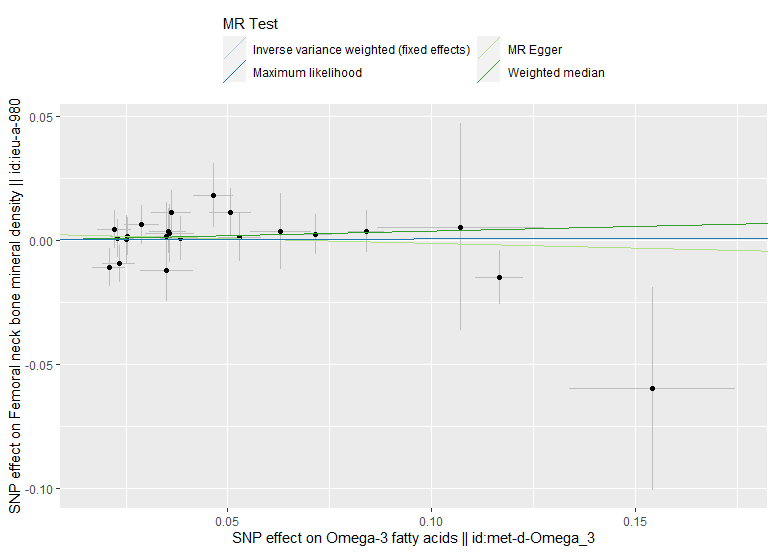


**Figure S14.** The scatter plot for omega-3 fatty acids on FN-BMD.


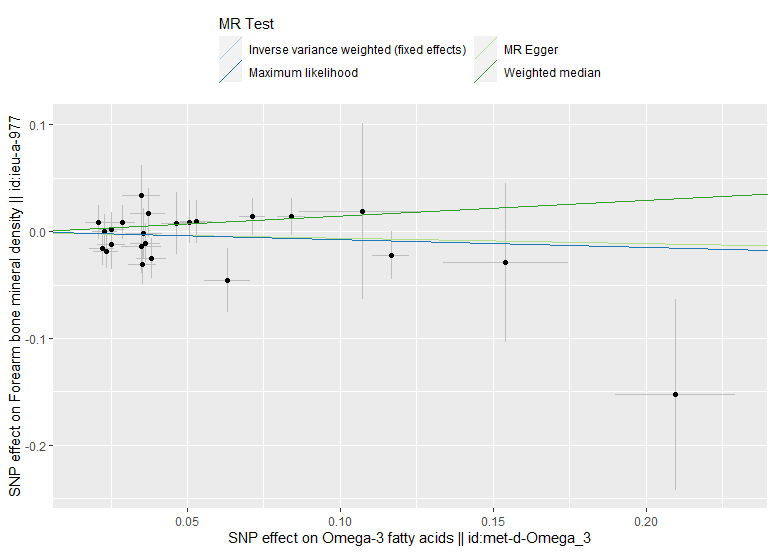


**Figure S15.** The scatter plot for omega-3 fatty acids on FA-BMD.


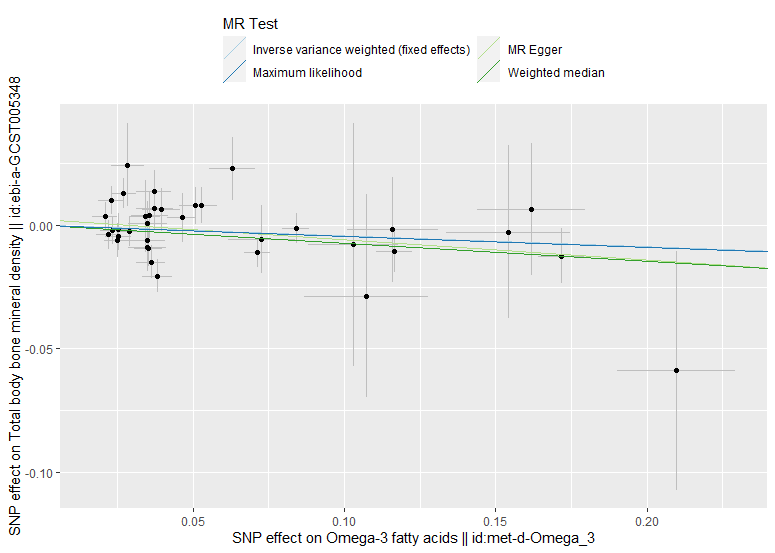


**Figure S16.** The scatter plot for omega-3 fatty acids on TB-BMD.


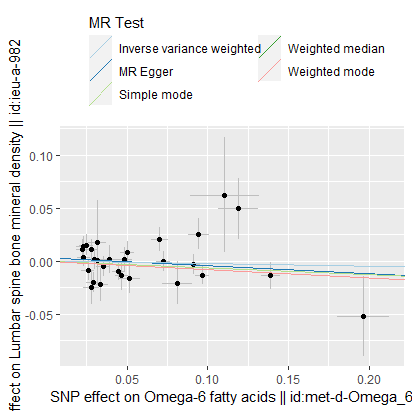


**Figure S17.** The scatter plot for omega-6 fatty acids on LS-BMD.


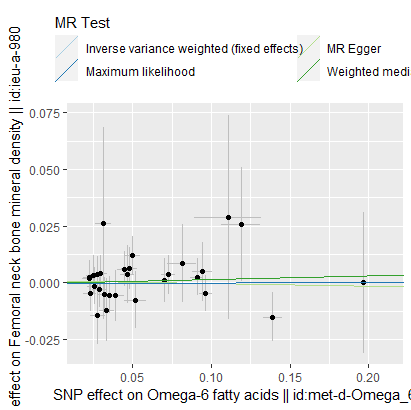


**Figure S18.** The scatter plot for omega-6 fatty acids on FN-BMD.


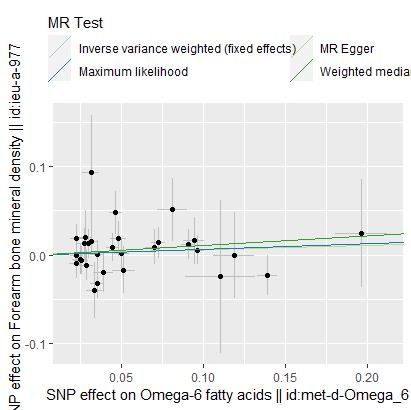


**Figure S19.** The scatter plot for omega-6 fatty acids on FA-BMD.


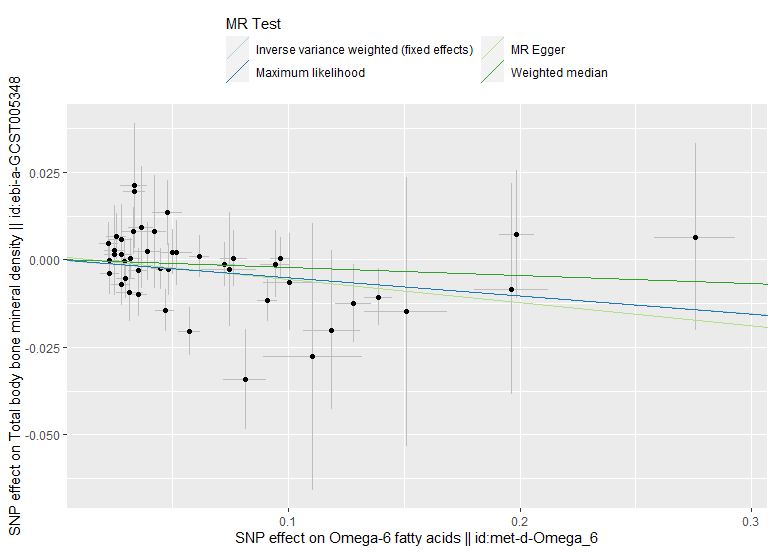


**Figure S20.** The scatter plot for omega-6 fatty acids on TB-BMD.


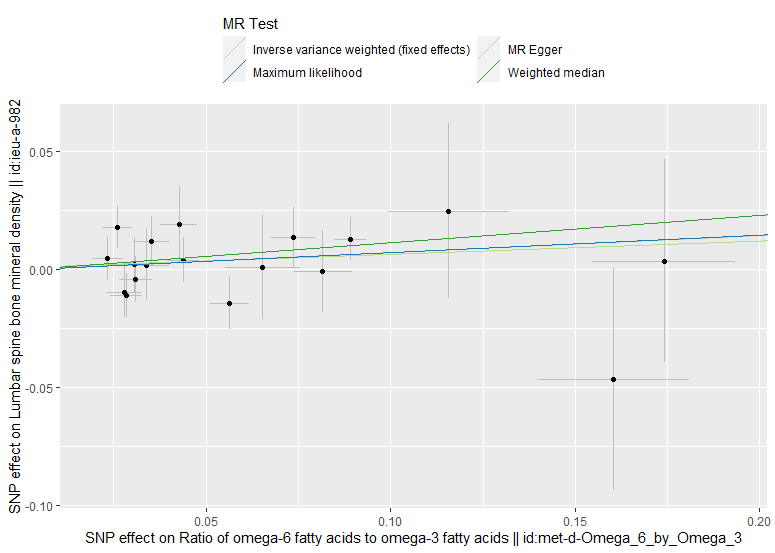


**Figure S21.** The scatter plot for Ratio of omega-6 fatty acids to omega-3 fatty acids on LS-BMD.


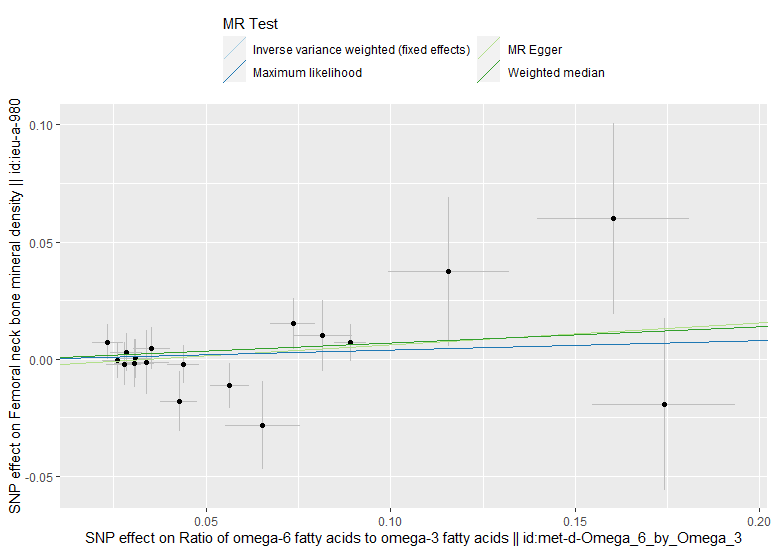


**Figure S22.** The scatter plot for Ratio of omega-6 fatty acids to omega-3 fatty acids on FN-BMD
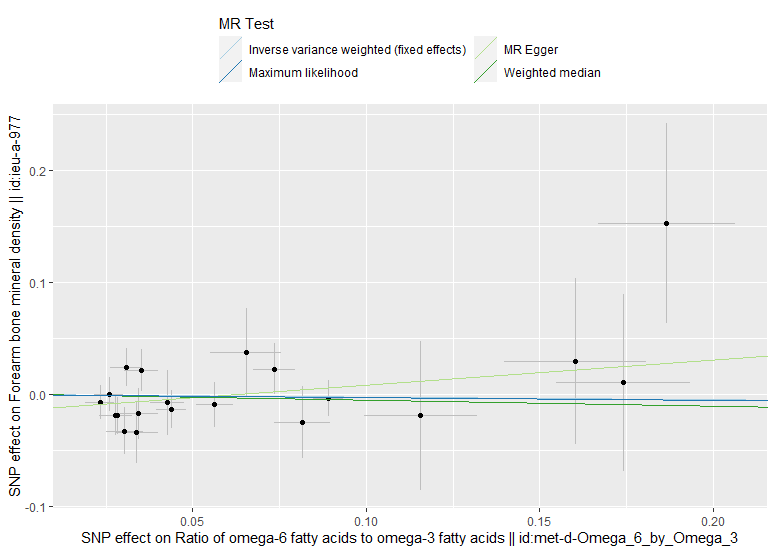
 **Figure S23.** The scatter plot for Ratio of omega-6 fatty acids to omega-3 fatty acids on FA-BMD.


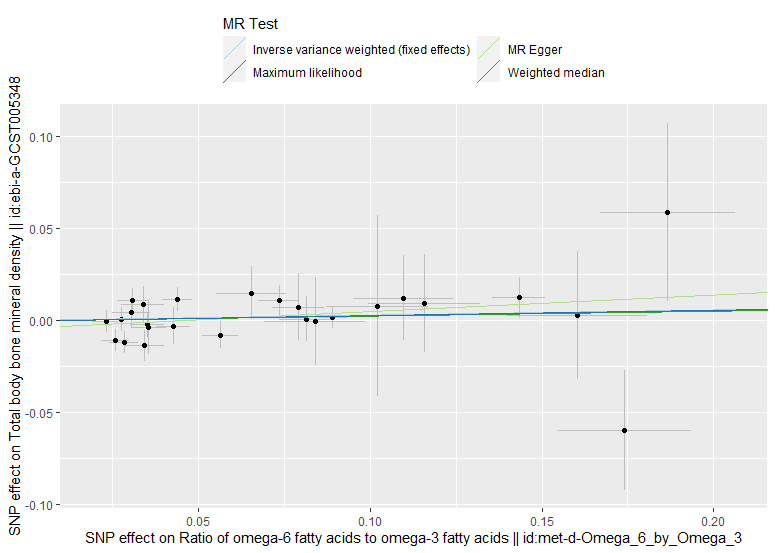


**Figure S24.** The scatter plot for Ratio of omega-6 fatty acids to omega-3 fatty acids on TB-BMD.
